# Amygdala–Ventral Striatum Functional Connectivity Underlies Craving in Gambling Disorder: A Mediating Role of Depressive Symptoms

**DOI:** 10.1111/adb.70065

**Published:** 2025-07-17

**Authors:** Yuzuki Ishikawa, Kentaro Katsuragi, Takahiko Inagaki, Kota Ebina, Yoshiteru Mutsuda, Morio Aki, Mami Shibata, Ayaka Hamamoto, Tsuyoshi Ando, Akihisa Iriki, Takashi Miyagi, Hiroto Mizuta, Ariyoshi Takemura, Takuro Murao, Hideaki Takeuchi, Ryosaku Kawada, Naoya Oishi, Hidehiko Takahashi, Toshiya Murai, Kosuke Tsurumi

**Affiliations:** ^1^ Department of Psychiatry, Graduate School of Medicine Kyoto University Kyoto Japan; ^2^ Ando Clinic Kyoto Japan; ^3^ Osaka Psychiatric Medical Center Osaka Japan; ^4^ Department of Psychiatry and Behavioral Sciences Institute of Science Tokyo Tokyo Japan; ^5^ Human Brain Research Center, Graduate School of Medicine Kyoto University Kyoto Japan; ^6^ Medical Institute of Developmental Disabilities Research Showa University Tokyo Japan; ^7^ Center for Brain Integration Research Institute of Science Tokyo Tokyo Japan

**Keywords:** amygdala, craving for gambling, depressive symptoms, gambling disorder, ventral striatum

## Abstract

Craving is an intense, strong urge to engage in addictive behaviours. Craving is supposed to be modulated by the connectivity between the amygdala and the ventral striatum (VS), a pivotal pathway for reward‐seeking behaviours. However, whether and how this connectivity underlies craving for gambling remains unclear, limiting our understanding of the pathophysiology of gambling disorder (GD). To address this issue, we analysed resting‐state functional connectivity (rs‐FC) between the amygdala and VS in 51 GD patients and 45 healthy participants. Craving for gambling was assessed using the Gambling Craving Scale (GACS). Among three GACS subscales, Desire exhibited a significant correlation with rs‐FC in the right amygdala–VS, while Anticipation and Relief showed no significant associations. Causal mediation analysis revealed that depressive symptoms significantly mediated the relationship between rs‐FC in the right amygdala–VS and Desire. These findings suggest that the amygdala–VS connectivity elicits the intense desire for gambling through negative emotions.

## Graphical abstract

Resting‐state functional connectivity (rs‐FC) between the right amygdala and the ventral striatum was associated with gambling craving. Of the subscales of the Gambling Craving Scale, only desire exhibited a significant correlation with amygdala‐ventral striatum (VS) rs‐FC. Further causal mediation analysis revealed that depressive symptoms mediated this association, suggesting that amygdala‐VS connectivity elicits an intense desire to gamble through negative emotions.

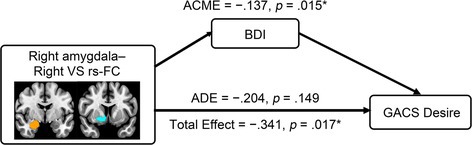

## 1 Introduction

Gambling disorder (GD) is a psychiatric disorder characterized by persistent gambling behaviour and its resulting negative consequences [1]. GD patients often exhibit craving, defined as a strong and intense urge to engage in addictive behaviours [1, 2]. Craving encompasses both the desire for positive rewards and escape from the problems caused by addiction [3], and it plays a pivotal role in substance‐related and addictive disorders such as cocaine use disorder, alcohol use disorder, tobacco use disorder and internet gaming disorder [4, 5, 6, 7]. Craving for gambling is considered a multidimensional process consisting of Anticipation (perceiving gambling as enjoyable), Desire (intense urge to gamble) and Relief (escape from negative states) similar to those observed in craving for cocaine and cigarettes [2, 5, 8]. In GD, craving has been associated with severity, loss aversion and emotional states [9, 10, 11] and has been identified as a risk factor for criminal behaviour and suicidal ideation [12, 13]. Despite its clinical importance, the neurobiological mechanisms underlying craving for gambling remain inadequately understood, limiting our understanding of the pathophysiology of GD for intervention and better prevention.

Functional connectivity between the amygdala and the ventral striatum (VS) may serve as a neurobiological basis for the craving for gambling. Anatomically, the basolateral complex of the amygdala has direct excitatory projections primarily to the ipsilateral VS. [14] This pathway has long been correlated with stimulus–reward associations, including reward‐seeking behaviours [15, 16, 17, 18, 19]. Previous research has shown that potentiation of the amygdala–VS connection facilitates cue‐triggered reward‐seeking behaviour, while attenuation of the connection is associated with reduced cue‐evoked sucrose intake [19]. Human task functional MRI studies have also reported the role of the amygdala and VS in reward‐seeking behaviours in healthy participants as well as in pathological conditions, including GD and alcohol use disorder, suggesting increased activity and connectivity in reward‐related contexts [20, 21, 22]. These findings suggest that the amygdala–VS pathway is associated with the dysfunction of the reward system in humans. However, no studies have examined how the amygdala–VS pathway is linked to craving for gambling in the absence of explicit cues or rewards. Resting‐state functional connectivity (rs‐FC) may reflect spontaneous synchrony of the amygdala–VS pathway in such contexts, potentially capturing the neural basis for craving to gamble. Furthermore, despite the multidimensional feature of craving, it remains unclear which aspects of craving are associated with the amygdala–VS pathway. Identifying the precise relationship between craving subscales and rs‐FC in the amygdala–VS may contribute to a characterization of the abnormal neural mechanisms of GD.

Craving is often suggested as being associated with depressive symptoms [2, 11, 23]. Gambling behaviour generally arises as an escape from everyday problems [24, 25, 26] and is driven by negative urgency—the tendency to act impulsively while in negative emotional states [7, 11]. Indeed, previous research predominantly supported depressive symptoms as a cause rather than a consequence of GD [27, 28, 29, 30]. These findings suggest that depressive symptoms may be a central component that elicits craving for gambling. Furthermore, animal studies have indicated that the amygdala–VS pathway underlies not only reward‐seeking behaviours but also emotional regulation, as excitation of this pathway has been shown to result in fear extinction and attenuation of stress‐related deficits [19, 31, 32, 33]. Considering these findings, depressive symptoms may play a mediating role in the relationship between the amygdala–VS pathway and craving for gambling. Exploring this mediating role could further our understanding of how depressive symptoms contribute to the pathophysiology of GD.

In this study, we hypothesize that rs‐FC between the amygdala and VS is associated with craving for gambling. Furthermore, we propose that depressive symptoms may mediate the link between rs‐FC in the amygdala–VS pathway and craving for gambling, thereby possibly advancing our understanding of the neurobiological mechanisms underlying craving for gambling.

## 2 Methods

### 2.1 Participants

Fifty‐seven Japanese GD patients and 61 Japanese healthy participants were recruited for the current study. Thirty‐seven patients were recruited from a treatment facility for GD, and 20 patients were recruited from Kyoto University Hospital and other hospitals and clinics. Healthy participants were recruited via word‐of‐mouth. All participants provided written informed consent to participate in the study. After excluding patients with missing data, 51 GD patients (aged 20–61 years; 48 men, 3 women) and 45 healthy participants (aged 22–55 years; 41 men, 4 women) were analysed in the current study (see Sections 2.2 and 2.3). All patients were abstinent from gambling and were under treatment for GD at the time of recruitment. GD symptoms were investigated using the Structured Clinical Interview for Pathological Gambling [34], and all patients met the DSM‐5‐TR criteria for GD. Comorbid disorders were assessed using the Structured Clinical Interview for Diagnostic and Statistical Manual of Mental Disorders Fourth or Fifth Edition [35, 36]. Comorbidities included a history of alcohol use disorder (AUD; *N* = 2), depression (N = 2), anxiety disorder (*N* = 2; comorbid with AUD or depression), and obsessive‐compulsive disorder (*N* = 1), as well as a current diagnosis of depression with antidepressant use (*N* = 4). None of the participants had a history of neurological injury or disease. Detailed demographic data are shown in Table 1.

**TABLE 1:** Demographic and clinical characteristics of participants in this study.

Characteristics	GD (*N* = 51)	Healthy participants (*N* = 45)	Statistics	*p* value
Sex (M/F)	48/3	41/4	*χ* ^2^ = 0.03	0.863
Age (months), mean (SD)	458.4 (129.0)	444.8 (113.1)	*t* = 0.55	0.584
Scanner (Trio/Verio)	35/16	24/21	*χ* ^2^ = 1.76	0.185
**Clinical scale, mean (SD)**				
GACS Anticipate	12.22 (4.67)	8.58 (4.32)	*t* = 3.96	< 0.001*
GACS Desire	6.92 (3.80)	3.53 (1.20)	*t* = 6.04	< 0.001*
GACS Relief	6.59 (4.04)	4.47 (2.20)	*t* = 3.24	0.002*
BDI	18.92 (11.18)	4.84 (4.38)	*t* = 8.30	< 0.001*
FTND	2.86 (2.43)	0.11 (0.61)	*t* = 7.83	< 0.001*
AUDIT	4.27 (5.42)	4.93 (4.89)	*t* = −0.63	0.533

Abbreviations: AUDIT, Alcohol Use Disorders Identification Test; BDI, Beck Depression Inventory; FTND, Fagerström Test for Nicotine Dependence; GACS, Gambling Craving Scale; GD, gambling disorder.

* *p* < 0.05.

### 2.2 Clinical Data

Craving for gambling was assessed using the Gambling Craving Scale (GACS) [2]. The GACS items are rated on a 1–7 Likert scale, with higher scores indicating a higher level of craving. Following a previous factor analysis [2], three subscales of GACS (Anticipation, Desire and Relief) were calculated as the sum of the corresponding items. Depressive symptoms were evaluated using the Beck Depression Inventory‐II (BDI‐II) [37]. The levels of cigarette smoking and alcohol use were assessed using the Fagerström Test for Nicotine Dependence (FTND) [38] and the Alcohol Use Disorders Identification Test (AUDIT) [39], respectively. Four GD patients and 16 healthy participants had missing clinical data and were thus excluded from further analysis.

### 2.3 MRI Acquisition

MRI data were acquired at a magnetic field strength of 3 T. A MAGNETOM Tim Trio or a MAGNETOM Verio scanner (Siemens Healthineers, Erlangen, Germany) was used at Kyoto University. We collected a T1‐weighted structural image, 10‐min eyes‐open resting‐state functional MRI (rs‐fMRI) images, as well as fieldmap images (magnitude and phase differences). T1‐weighted brain images were acquired using three‐dimensional magnetization‐prepared rapid acquisition with a gradient echo (3D‐MPRAGE) with the following parameters: TR = 2000 ms, TE = 3.40 ms, TI = 990 ms, FOV = 225 × 240 mm, matrix size = 240 × 256, spatial resolution = 0.9375 × 0.9375 × 1.0 mm. Rs‐fMRI images were acquired using a single‐shot gradient‐echo echo‐planar imaging with the following parameters: TR = 2500 ms, TE = 30 ms, flip angle = 80°, FOV = 212 × 212 mm, matrix size = 64 × 64, spatial resolution = 3.3125 × 3.3125 × 3.2 mm, slice gap = 0.8 mm, number of volumes = 240. During rs‐fMRI scanning, participants were instructed to look at a fixation point while not thinking of anything. Fieldmap images were acquired with a double‐echo spoiled gradient‐echo sequence with the following parameters: TR = 488 ms, TE = 4.92/7.38 ms, spatial resolution = 3.3125 × 3.3125 × 3.2 mm, slice gap = 0.8 mm, flip angle = 60°. Two GD patients were excluded due to missing MRI data.

### 2.4 MRI Preprocessing

The FMRIB Software Library (FSL) 6.0.7.1, FSL FIX 1.06.15, FreeSurfer 7.4.1, MATLAB 2023a, SPM 12 r7771, and CONN 22.a were used for the following preprocessing. Structural images underwent bias‐field correction, segmentation by fsl‐anat and skull‐stripping. Rs‐fMRI images underwent motion correction by MCFLIRT, bias‐field correction by fsl‐anat and skull‐stripping by synthstrip in FreeSurfer. Magnitude and phase images were used to create a fieldmap image by the fsl_prepare_fieldmap function. Slice timing correction, distortion correction, and temporal high‐pass filtering were performed using FEAT in FSL. Independent component analysis (ICA) was applied to rs‐fMRI images using MELODIC in FSL, followed by ICA‐based denoising by FIX. To minimize the effect of motion, framewise displacement (FD) was calculated based on the rs‐fMRI motion parameters, and subjects with > 0.5 mm FD in more than 20% of volumes were to be excluded. However, no participants were excluded according to this criterion. Using the CONN toolbox, FIX‐denoised rs‐fMRI images underwent normalization to MNI space, ART‐based scrubbing, spatial smoothing using a Gaussian kernel with a full‐width‐at‐half‐maximum of 8 mm, band‐pass filtering with a frequency window of 0.01–0.1 Hz and denoising using the anatomical CompCor with the five largest principal components of white matter and cerebrospinal fluid, as well as translational and rotational movement parameters and their first‐order temporal derivatives as regressors.

### 2.5 Functional Connectivity

We used an amygdala template based on FreeSurfer's automatic labelling [40, 41] and a VS atlas based on the anatomy‐guided labelling of MRI images [42]. BOLD time courses of the unilateral amygdala and VS were extracted from denoised, unsmoothed rs‐fMRI images preprocessed in the CONN toolbox. Pearson's correlation coefficients between ipsilateral BOLD time courses of the amygdala and VS were calculated and *z*‐transformed.

### 2.6 Statistical Analysis

#### 2.6.1 Demographic and Clinical Data

The mean and standard deviation for continuous variables, as well as the number and ratio of categorical variables, were calculated. *T* tests and chi‐squared tests were used to compare the differences between the GD patients and healthy participants. Statistical significance was set as *p* < 0.05.

#### 2.6.2 Harmonizing Scanner Differences

ComBat harmonization was used to adjust for scanner effects using pycombat 0.20 in Python. Other covariates (i.e., age, sex, FTND and AUDIT) were integrated into the batch‐effect estimation to preserve their effects.

#### 2.6.3 Association Between Functional Connectivity and Craving for Gambling

Partial correlation analysis was conducted to examine the association between rs‐FC in the amygdala–VS and GACS subscales. Age, sex, FTND and AUDIT were included as covariates. Partial correlation analyses were performed for ipsilateral rs‐FC pairs and GACS subscales, applying Bonferroni correction for all comparisons (two rs‐FC pairs × three subscales = six comparisons). Rs‐FC and GACS subscales showing significant associations were used for further analysis. Statistical significance was set as *p* < 0.05.

#### 2.6.4 Causal Mediation Analysis

To test whether depressive symptoms mediate the link between rs‐FC in the amygdala–VS and craving for gambling, causal mediation analysis was performed. We set rs‐FC, BDI and GACS subscales as exposure, mediator and outcome, respectively, as presented in Section 1. Causal mediation analysis was conducted using the mediation package 4.5.0 in R 4.3.2. The total effect, average direct effect (ADE) and average causal mediated effect (ACME), as well as their 95% credible intervals and *p* values were estimated with 10 000 Monte Carlo simulations. Age, sex, FTND and AUDIT were used as covariates. Statistical significance was set as *p* < 0.05.

#### 2.6.5 Group‐Level Comparison of Functional Connectivity

To test the between‐group difference in the amygdala–VS rs‐FC, analysis of covariance was performed using pingouin 0.5.5 in Python with age, sex, FTND, and AUDIT as covariates. Statistical significance was set as *p* < 0.05.

## 3 Results

### 3.1 Demographics and Clinical Assessments

Sex, age and the scanner ratio were not significantly different between GD patients and healthy participants. All GACS subscales, BDI and FTND scores in GD patients were significantly higher than those in healthy participants (Table 1). The AUDIT score was not significantly different between the study groups.

### 3.2 Association Between GACS and Functional Connectivity in the Amygdala–VS

**FIGURE 1:**
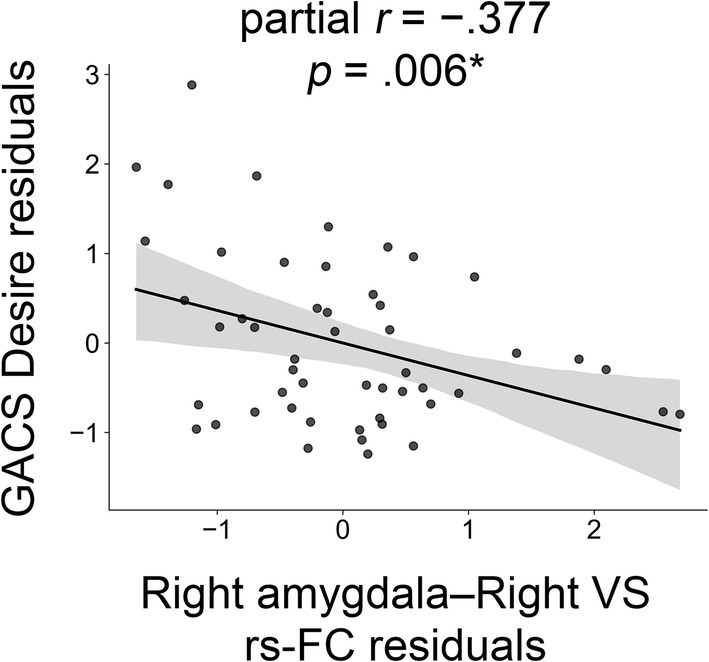
Association between GACS Desire and amygdala–ventral striatum functional connectivity. GACS, Gambling Craving Scale; VS, ventral striatum; FC, functional connectivity. *p < 0.05.

Rs‐FC in the right amygdala–right VS was significantly correlated with GACS Desire (*ρ* = −0.377, 95% CI = [−0.591, −0.113], *p* = 0.006) in GD patients (Figure 1). Other rs‐FC values did not exhibit significant correlations with any of the GACS subscales. Right amygdala‐right VS connectivity was significantly associated with BDI scores (*ρ* = −0.390, 95% CI = [−0.616, −0.130], *p* = 0.005) (Figure S1).

**FIGURE 2:**
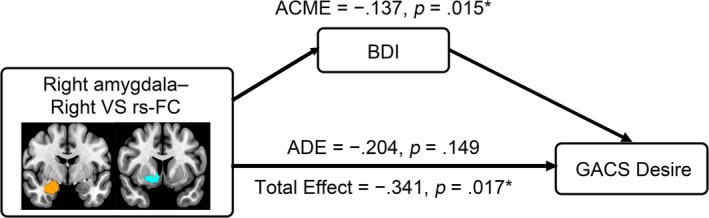
Associations between GACS Desire, BDI and amygdala–ventral striatum functional connectivity. BDI mediates the causal relationship between rs‐FC in the right amygdala–right VS and GACS Desire. VS, ventral striatum; FC, functional connectivity; GACS, Gambling Craving Scale; BDI, Beck Depression Inventory; ACME, average causal mediated effect; ADE, average direct effect. *p < 0.05.

Our mediation model, including rs‐FC in the right amygdala–right VS as exposure, BDI as mediator, and GACS Desire as outcome, demonstrated a significant total effect (−0.341, 95% CI = [−0.617, −0.069], *p* = 0.017) and ACME (−0.137, 95% CI = [−0.306, −0.018], *p* = 0.015) in GD patients (Figure 2). ADE was not significant (−0.233, 95% CI = [−0.480, 0.076], *p* = 0.149). The proportion of the mediated effect was 0.389 (95% CI = [0.037, 1.506], *p* = 0.031).

Sensitivity analysis restricted to male participants (*N* = 48) showed that the correlation between right amygdala–right VS rs‐FC and GACS Desire remained significant (*ρ* = −0.353, 95% CI = [−0.634, −0.070], *p* = 0.016), whereas the indirect effect was not significant (ACME = −0.110, 95% CI = [−0.278, 0.010], *p* = 0.069).

### 3.3 Comparison of Rs‐FC With Healthy Participants

No significant difference in rs‐FC in the right amygdala–right VS was found between GD patients and healthy participants (*F*[1, 297] = 0.940, *p* = 0.334, *d* = 0.269).

## 4 Discussion

This study investigated the neural mechanisms of craving for gambling and their potential causal relationships in GD patients. Among the three subscales of the GACS, Desire exhibited a significant correlation with rs‐FC in the right amygdala–VS. We tested the causal mediation model, in which depressive symptoms mediate the causal relationship between rs‐FC in the right amygdala–VS and GACS Desire. This model showed significant total and indirect effects, with an insignificant direct effect. The right amygdala–VS rs‐FC did not show significant differences between GD patients and healthy participants. By demonstrating that craving is associated with amygdala–ventral striatum connectivity and that depressive symptoms mediate this link, the present study clarifies the pathway through which the desire for gambling emerges, advancing our understanding of the underlying neural mechanisms.

The current study demonstrated that the Desire dimension, but not the Anticipation and Relief dimensions, was significantly correlated with rs‐FC in the amygdala–VS. This finding highlights the distinct characteristics of these craving subcomponents. While Desire reflects an intense and immediate urge, Anticipation and Relief may be more reflective of the belief that gambling will lead to positive outcomes or alleviate negative emotional states [2]. Unlike Desire, Anticipation and Relief may involve more deliberative processes, requiring cognitive appraisal of the potential outcomes of gambling and their emotional implications. Our results suggest that amygdala–VS connectivity may be particularly associated with the emergence of desire, potentially triggering an intense and unstoppable urge to gamble. Given that the VS integrates signals from various brain regions to modulate reward‐related behaviours [43], projections from brain regions other than the amygdala may be associated with the Anticipation and Relief dimensions of the craving for gambling. Previous studies have highlighted the importance of prefrontal modulation of the striatum in cognitive regulation of craving [44, 45]. Future studies investigating the interplay between prefrontal regions, the VS and the amygdala may provide further insights into the neural mechanisms underlying the multidimensional nature of craving for gambling.

The current study found a negative correlation between GACS Desire and rs‐FC in amygdala–VS, indicating that lower rs‐FC is associated with a stronger desire to gamble. In contrast, previous research on human task fMRI exhibited higher connectivity in GD patients than in healthy participants, suggesting the greater engagement of the amygdala–VS pathway in cue‐evoked states in GD [21]. Similarly, several animal studies have reported that excitation in the amygdala–VS pathway promotes reward‐seeking behaviours, which may alleviate negative emotional states such as long‐term fear [19, 32]. Taken together, craving and negative emotional states may involve higher amygdala–VS connectivity in reward‐related contexts and lower connectivity at rest compared with healthy participants. We speculate that this task‐rest divergence may underlie the mechanisms of addiction, which can be conceptualized as a feedback model. In this model, lower baseline rs‐FC in the amygdala–VS causes greater negative emotions, which drive an immediate urge for addictive behaviours. Upon engaging in these behaviours, amygdala–VS connectivity temporarily increases, leading to an immediate reduction in negative emotions. Repetition of this cycle may reinforce addictive behaviours as a means of regulating negative emotions. Therefore, aberrant amygdala–VS connectivity might result in a constant need for drugs or addictive behaviours to alleviate negative emotions. This model aligns with our finding that depressive symptoms mediate the relationship between rs‐FC in amygdala–VS and the Desire dimension of craving. Future research should examine this feedback model to elucidate the neural mechanisms of GD.

The current study found no significant differences in the right amygdala–VS rs‐FC between GD patients and healthy participants. While this study found no such group‐level difference at rest, previous research revealed significantly greater amygdala–VS coupling during the delay and probability discounting task [21]. This difference indicates that increased task‐evoked connectivity, or a greater task–rest difference in the amygdala–VS, may characterize the abnormality in GD rather than the baseline level of synchrony itself. We did not extend the mediation analysis to healthy participants because their GACS and BDI scores clustered at floor level, leaving virtually no variance for path estimation. Future studies with larger samples are needed to confirm whether the same pathways operate in non‐clinical populations.

The current findings highlight the importance of depressive symptoms in their mediating role in the link between amygdala–VS rs‐FC and craving, as well as the possibility of effective intervention of negative emotional states and underlying neural mechanisms to suppress craving for gambling. Pharmacological intervention may be able to target this specific pathway or neurotransmitter systems responsible for the amygdala–VS connection. Although several studies have investigated pharmacological therapies for GD targeting serotonergic and dopaminergic systems, their efficacy has not been fully established [46, 47]. Considering the findings that the D1‐type receptor could modulate the reinforcing effect of amygdala–VS on reward‐seeking behaviour through glutamatergic release from the amygdala [19], a pharmacological intervention targeting the dopaminergic system may be promising. Furthermore, neuromodulation studies showed that repetitive transcranial magnetic stimulation (rTMS) on the dorsolateral prefrontal cortex reduced craving in GD [48]. Additionally, recent research showed that cue‐induced craving increases with stronger amygdala–VMPFC coupling and proposed that VMPFC‐targeted rTMS may modulate a fronto‐limbic circuit involving the amygdala, ventral striatum, and anterior cingulate cortex [ 49]. Together with findings that the striatum acts as a hub integrating inputs from both regions [ 43], these studies support the notion that prefrontal–amygdala–striatal dynamics may underlie mechanisms of craving suppression and recovery in addiction. Future studies should characterize the detailed role of the prefrontal cortex, its interaction with the amygdala–VS pathway, and the neurobiological mechanisms of the rTMS action in craving for gambling.

There are several limitations to this study. First, because mediation analysis does not establish causality but rather assumes it, and because the cross‐sectional design of this study prevented collection of the clinical and MRI measures in the temporal sequence required for causal inference, the mediation findings should be interpreted with caution. Second, although the odds ratio for pathological gambling among males compared with females is approximately 4.4 [50], the 51 GD patients included only three females. Sensitivity analysis restricted to male participants showed a similar trend but failed to reach significance, due to reduced sample size and the possibility of stronger effects in female patients, underscoring the need for future sex‐balanced studies. Lastly, the patients in this study were under treatment for GD, limiting its generalizability to treatment‐naïve and non‐treatment‐seeking populations.

In summary, the present study found an association between amygdala–ventral striatum rs‐FC and craving for gambling. Causal mediation analysis illustrated the potential mediating effect of depressive symptoms on craving. These results provide substantial evidence regarding craving in gambling disorder and should contribute to the development of effective interventions targeting the underlying neural mechanisms.

## Permission to Reproduce Material From Other Sources

No material was reproduced from other sources.

## Author Contributions

**Yuzuki Ishikawa:** conceptualization, methodology, software, formal analysis, investigation, data curation, writing – original draft, writing – review and editing, visualization. **Kentaro Katsuragi:** investigation, resources, data curation, writing – review and editing, project administration. **Takahiko Inagaki:** investigation, writing – review and editing. **Kota Ebina:** investigation, writing – review and editing. **Yoshiteru Mutsuda:** investigation, writing – review and editing. **Morio Aki:** investigation, writing – review and editing. **Mami Shibata:** investigation, resources, writing – review and editing. **Ayaka Hamamoto:** investigation, writing – review and editing. **Tsuyoshi Ando:** investigation, resources, writing – review and editing. **Akihisa Iriki:** investigation, resources, writing – review and editing. **Takashi Miyagi:** investigation, resources, writing – review and editing, project administration. **Hiroto Mizuta:** investigation, resources, writing – review and editing. **Ariyoshi Takemura:** investigation, resources, writing – review and editing. **Takuro Murao:** investigation, resources, writing – review and editing. **Hideaki Takeuchi:** investigation, resources, writing – review and editing, project administration. **Ryosaku Kawada:** investigation, resources, writing – review and editing, project administration. **Naoya Oishi:** software, writing – review and editing. **Hidehiko Takahashi:** writing – review and editing, funding acquisition. **Toshiya Murai:** supervision, writing – review and editing, funding acquisition. **Kosuke Tsurumi:** conceptualization, methodology, investigation, resources, project administration, writing – review and editing, supervision, funding acquisition.

## Ethics Statement

All methods reported in the current study were performed in accordance with the Ethical Guidelines for Medical and Biological Research Involving Human Subjects. The approval of the current study was obtained from Kyoto University Graduate School and Faculty of Medicine Ethics Committee (registration number R0999).

## Consent

All participants submitted their written informed consent to participate prior to their recruitment.

## Conflicts of Interest

The authors declare no conflicts of interest.

## Supporting information


**Figure S1** **Association between BDI scores and amygdala–ventral striatum functional connectivity**. BDI Beck Depression Inventory, VS ventral striatum, FC functional connectivity. *: *p* < 0.05.

## Data Availability

Data supporting the findings of this study are available from the corresponding author upon reasonable request. The data are not publicly available because they contain information that could compromise the privacy of the research participants.
