# Supplementary material for: Amygdala–Ventral Striatum Functional Connectivity Underlies Craving in Gambling Disorder: A Mediating Role of Depressive Symptoms
Source: Addict Biol. 2025 Jul 17;30(7):e70065. doi: 10.1111/adb.70065 (PMC12268111; doi:10.1111/adb.70065)
Supplement: Supplementary file 1 — Figure S1 Association between BDI scores and amygdala–ventral striatum functional connectivity. BDI Beck Depression Inventory, VS ventral striatum, FC functional connectivity. *: p < 0.05. [file ADB-30-e70065-s001.docx]

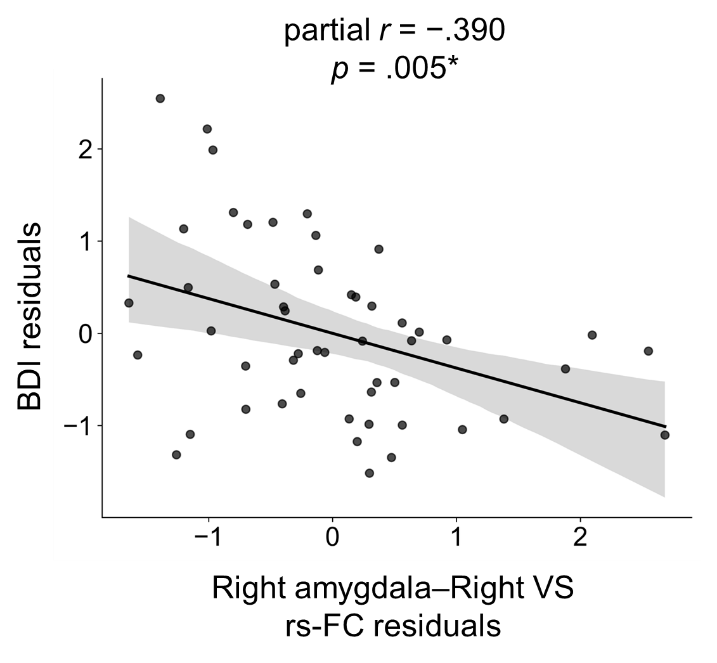


**Supplementary Figure 1. Association between BDI scores and amygdala–ventral striatum functional connectivity**. BDI Beck Depression Inventory, VS ventral striatum, FC functional connectivity. *: *p* < 0.05.
